# Genomics costing tool: considerations for improving cost-efficiencies through cross scenario comparison

**DOI:** 10.3389/fpubh.2024.1498094

**Published:** 2025-01-15

**Authors:** Marco Marklewitz, Alexandr Jaguparov, Aude Wilhelm, Oluwatosin Wuraola Akande, Biran Musul, Angela Lee Poates, Babak Afrough, Ashley Norberg, Noah Clayton Hull, Soudeh Ehsani, Group members of GCT pilot working group, Joanna Salvi Le Garrec, Toni Whistler

**Affiliations:** ^1^FIND, Geneva, Switzerland; ^2^Infectious Hazard Management, World Health Organization Regional Office for Europe, Copenhagen, Denmark; ^3^New Variant Assessment Platform, UK Health Security Agency, London, United Kingdom; ^4^Department of Epidemic and Pandemic Preparedness and Prevention, World Health Organization, Geneva, Switzerland; ^5^Global Health, Association of Public Health Laboratories, Bethesda, MD, United States; ^6^Joint Infectious Diseases Unit, World Health Organization Regional Office for Europe, Copenhagen, Denmark; ^7^Technical Advice and Partnership Department, The Global Fund to Fight AIDS, Tuberculosis and Malaria, Geneva, Switzerland

**Keywords:** next-generation sequencing, costing tool, COVID-19, cost-analysis, genomic surveillance

## Abstract

Next-generation sequencing (NGS) is crucial for monitoring and investigating infectious disease outbreaks, providing essential data for public health decisions. The COVID-19 pandemic has significantly expanded pathogen sequencing and bioinformatics capacities worldwide, creating an opportunity to leverage these advancements for other pathogens with pandemic and epidemic potential. In response to the need for a systematic cost estimation approach for sustainable genomic surveillance, particularly in low- and middle-income countries, five institutions collaborated to develop the genomics costing tool (GCT). These institutions are the Association of Public Health Laboratories (APHL), FIND, The Global Fund to Fight AIDS, Tuberculosis and Malaria, the UK Health Security Agency (UKHSA), and the World Health Organization (WHO). To validate the GCT, it was piloted in public health laboratories across three WHO regions: African, Eastern Mediterranean, and European. The pilot exercises were intended to assess the tool’s accuracy, utility, and functionality, exploring scenarios for validating past expenditure, routine use, cost optimization, and scaling up sequencing services. Data from these pilots demonstrated significant cost reductions per sample with increased throughput, underscoring the economic benefits of the optimized use of sequencing platforms underpinned by sample throughput. The GCT enables laboratories to estimate and visualize costs, plan budgets, and improve cost-efficiencies for sequencing and bioinformatics based on factors such as equipment purchase and preventative maintenance, reagents and consumables, annual sample throughput, human resources training, quality assurance and management. This publication shares key findings from pilot exercises offering detailed insights into the cost of routine NGS implementation using either short- or long-read sequencing technologies, demonstrating the utility of GCT as an asset to support efforts for sustainable funding and strategic planning in genomic surveillance.

## 1 Introduction

Next-generation sequencing (NGS) is essential for pathogen surveillance and outbreak investigation, contributing to evidence-based decision making and appropriate public health actions. NGS is instrumental in identifying, characterizing, and monitoring pathogen evolution and transmission patterns, as well as in the development of diagnostics, therapeutics and vaccines (1, 2). The COVID-19 pandemic led to an unprecedented increase in countries’ interest and capacity for sequencing and bioinformatics for severe acute respiratory syndrome coronavirus 2 (SARS-CoV-2) (1). Since then, several global recommendations, including a resolution from the World Health Assembly (3, 4), and the Global genomic surveillance strategy for pathogens with pandemic and epidemic potential, 2022–2032 (5), have urged countries to leverage this expanded sequencing capacity to other pathogens of public health relevance.

Access to timely genomic sequencing requires sustainable funding (1, 6). While the cost of genomic sequencing has steadily decreased (7, 8), it remains significant, particularly for low- and middle-income countries. To support genomic sequencing, relevant stakeholders need to understand the costs associated with implementing sequencing across the genomic surveillance value chain. This includes costs for biosafety/biosecurity, infrastructure, reagents and consumables, workforce as well as quality assurance and management components.

In response to country requests and its recognized value, a genomics costing tool (GCT) was co-developed by five institutions: Association of Public Health Laboratories, FIND, The Global Fund to Fight AIDS, Tuberculosis and Malaria, UK Health Security Agency UK (HSA), and the World Health Organization (WHO). The first version of the GCT was launched in December 2023, to support short- and long-term financial planning and budgeting for SARS-CoV-2 genomic sequencing. The tool calculates total costs for establishing and operating a sequencing laboratory, providing estimates of both overall cost per sample, broken down by distinct expense categories (e.g., equipment, personnel, and training) and workflow steps (9). The total establishment cost includes annual reagent and consumable, recommended equipment procurement (including first-year maintenance and calibration), bioinformatics infrastructure, annual personnel salary and training, annual costs related to laboratory facilities and transportation, and annual quality management system expenditures. The operational costs and the cost per sample consider the same costs except equipment, where annual equipment amortization, maintenance and calibration cost is calculated instead of procurement cost. The GCT includes initial investment costs, such as equipment procurement and associated maintenance, as critical input parameters. By proportionally integrating these costs into per-sample calculations, the tool enables laboratories to assess economic feasibility in a manner tailored to their specific operational contexts. A comprehensive understanding of the financial implications of NGS implementation can offer valuable insights for programs aiming to improve their workflow efficiency or secure the necessary funding to sustain a genomic surveillance program.

As part of the GCT development process, the tool was piloted between June and August 2023 in three national public health laboratories across different WHO regions. The objectives of the pilot were to: (1) assess the accuracy, utility, and functionality of the GCT, (2) validate and refine the tool based on the initial findings, and (3) gather feedback on the tool’s perceived usability and value. This paper describes insights obtained from three implementation scenarios on the detailed costs of routine sequencing for pathogen genomic surveillance.

## 2 Materials and methods

The development of the GCT, including its rationale and utility for countries, has been described previously (9).

### 2.1 Setting

The GCT was piloted in national public health laboratories in three WHO Regions: African, Eastern Mediterranean, and European. The selection of laboratories was based on criteria aimed at validating the tool across diverse contexts, to establish its utility and relevance to a broad range of potential users. These criteria included annual throughput (varying sequencing volumes to test the GCT’s adaptability to different scales of operation), sequencing platforms (a mix of laboratories with different platforms to assess compatibility across technologies), geographical location (diverse socioeconomic contexts), and country income classification (to evaluate the tool’s relevance across different resource settings). Table 1 provides additional details on the selected laboratories. While the GCT is designed for various laboratories, both public and private, the pilot exercises focused on national public health laboratories, which deliver timely and reliable results primarily aimed at disease control and prevention.

**Table 1:** Contextual information on the three national public health laboratories that participated in the pilot exercises.

	Laboratory 1	Laboratory 2	Laboratory 3
World Health Organization region	European	African	Eastern Mediterranean
Population^1^	7,100,800	34,121,985	4,644,384
Pilot country income level^2^	Lower-middle income	Lower-middle income	High-income economy
Mode of pilot exercise	Onsite	Onsite	Virtual
Date of pilot exercise (Duration)	June 2023 (3 days)	July 2023 (3 days)	August 2023 (2 days)
No. of pilot exercise participants	3	8	8
Job function of participants	Laboratory director, laboratory technicians	Head of laboratory, laboratory technicians, research assistants, laboratory manager, bioinformatician	Laboratory director, laboratory technicians, bioinformaticians, procurement staff, quality manager
Annual throughput in 2022 (SARS-CoV-2)	260	2,500	600

1 World Bank population data 2023: https://data.worldbank.org/indicator/SP.POP.TOTL?locations=GH-KG-OM.

2 World Bank country and lending groups, fiscal year 2024: https://datahelpdesk.worldbank.org/knowledgebase/articles/906519-world-bank-country-and-lending-groups.

### 2.2 Pre-pilot activities

Approximately 1 month before the pilot exercises, each participating laboratory was asked to complete a pre-pilot survey (Supplementary file 1). This survey was designed to understand the laboratory’s costing needs and gather the minimum data required for the pilot exercise. The survey collected information on the laboratory profile, sequenced pathogens, and sequencing capacities including sequencing platforms, annual sample throughput, laboratory infrastructure, human resources, training, and finances. To further assist laboratories in preparing for the pilot exercise, the survey also included a list of necessary input data needed for the tool during the pilot exercise.

On receiving the completed surveys, the pilot team reviewed the responses and prepared for the pilot visit, which included the development of relevant costing exercises.

### 2.3 Pilot exercises

Pilot exercises were conducted over 2 or 3 days, either in-person or virtually, with the support of at least two members of the GCT pilot working group. These exercises included a multidisciplinary group of participants, including laboratory directors, laboratory technicians, bioinformaticians, procurement personnel, and quality managers (Table 1). This composition ensured diverse perspectives and comprehensive input during the pilot activities.

Each pilot exercise followed a structured format. Beginning with an introduction to the activity objectives, providing participants with a clear understanding of the scope of tool, and validation procedure to inform final pre-launch GCT revisions. This was followed by a discussion of the pre-pilot survey, which aimed to collect baseline information about the laboratory’s sequencing and bioinformatics infrastructure (Supplementary file 1). In addition, missing information, and an overview of the costing scenarios were discussed. Thereafter, participants were introduced to the tool, including a detailed demonstration of its structure and functionality.

At each of the participating laboratories, three different costing scenarios were explored using data from their laboratory. The first scenario involved a validation analysis to help countries verify the cost of sequencing and bioinformatics activities from the previous year. The second focused on cost optimization, assisting countries in making informed decisions to optimize sequencing workflows for better value. The third was a scale-up scenario, intended to help build an investment case by estimating the cost of expanding sequencing and bioinformatics services.

Throughout the exercise, participants engaged in hands-on use of the GCT, supported by the pilot working group to ensure accuracy and address any technical challenges. After each pilot exercise results from the scenarios were discussed, including a facilitated feedback session to capture participants’ experience, insights, and suggestions for improving the tool’s functionality and usability.

### 2.4 Post-pilot activities

To capture comprehensive feedback on the GCT, a post-pilot survey (Supplementary file 2) was created and administered to two of the pilot laboratories.

To best showcase the utility of the GCT in this manuscript, the input data gathered during the pilot exercises for each laboratory was extracted and compared in three potential costing scenarios. Analysis for the Validation Scenario used predefined parameters: an annual throughput of 600 samples with a single run performed each week over 36 work weeks, using the laboratory’s existing instrumentation and specific library preparation kit and sequencing reagents for SARS-CoV-2. Bioinformatic analysis was costed using in-house computing for low throughput situations. The Optimization Scenario used a different instrument with the same annual throughput as the Validation Scenario, while the Scale-up Scenario utilized the same instrument with a higher sample throughput (Tables 2, 3).

**Table 2:** Comparison of genomics costing tool (GCT) output data from laboratories in the WHO European and African regions for sequencing of SARS-CoV-2 using Illumina (Laboratory 1) and Oxford Nanopore Technology (ONT) (Laboratory 2) instruments under 3 different scenarios.

Costing output categories^b^	Laboratory 1 (European region / LMIC)			Laboratory 2 (Africa region / LMIC)		
	Validation	Optimization	Scale-up	Validation	Optimization	Scale-up
Initial establishment cost	536,761	484,867	531,665	265,962	331,637	272,180
Total first year costs for the laboratory	763,057	703,929	1,171,916	539,441	629,966	776,710
Annual operational cost	226,296	219,062	640,251	273,479	298,329	504,530
Annual reagent & consumable costs	73,564	80,601	487,519	55,958	55.958	287,008
Average cost/sample	377	365	128	456	497	101
Reagents and consumables /sample	123	134	98	93	93	57
Equipment maintenance & calibration cost/sample	224	200	27	246	288	30
Sequencer loading capacity/run	28%	35%	99%	17%	3%	72%

Laboratory 1 GCT inputs: Validation Scenario: An annual throughput of 600 samples with a single run being performed each week over 36 weeks, using an *Illumina MiSeq* and *EasySeq SARS-CoV-2 whole genome sequencing* (*WGS*) *Library Preparation Kit* (*96 samples*) plus the *MiSeq Reagent Kit v2* (*300 cycles*). Bioinformatic analyses were costed using in-house computing for low throughput situations with an assumed sequencing success rate of 80%^a^. Optimization Scenario: Using an *Illumina MiniSeq* instrument with the associated *MiniSeq High Output Reagent Kit* (*300 cycles*). All other inputs were the same as in the Validation Scenario above. Scenario C: Using the same instrument as in the Validation Scenario but with a higher sample throughput of 5,000 samples/year and 2 runs/week.

Laboratory 2 GCT inputs: changes to the scenarios from laboratory 1 were: the Validation and Scale-up Scenarios used ONT MinION instruments, the Optimization Scenario used the ONT GridION. All 3 scenarios were costed using the ONT bundled sequencing reagents with the COVID Mini library preparation kit. The assumed sequencing success rate was 90% for all ONT scenarios^a^.

a Sequencing success rate must be estimated by the user reflecting level of expertise and previous experience.

b All costs are shown in United States dollars (USD).

**Table 3:** Genomics costing tool output data calculated for three scenarios for Laboratory 3 in a high-income economy country from the WHO Eastern Mediterranean region for both Illumina and Oxford Nanopore Technology (ONT) instruments.

Costing output categories^b^	Laboratory 3 (Eastern Mediterranean / HIC)					
	Illumina instruments			ONT instruments		
	Validation	Optimization	Scale-up	Validation	Optimization	Scale-up
Initial establishment cost	738,890	660,073	745,799	628,723	542,777	635,632
Total first year costs for the laboratory	1,436,057	1,346,145	1,917,802	1,206,764	1,185,834	1,502,166
Annual operational cost	697,167	686,072	1,172,003	578,041	643,057	866,534
Annual reagent & consumable costs	159,974	169,924	629,810	100,041	100,041	388,534
Average cost/sample	1,162	1.143	234	963	1,072	173
Reagents and consumables /sample	258	283	125	166	166	77
Equipment maintenance & calibration cost/sample	186	143	22	79	188	10
Sequencer loading capacity/run	20%	25%	83%	13%	3%	52%

The GCT inputs for the Validation Scenario were an annual throughput of 600 samples with a single run being performed each week over 50 weeks, using an *Illumina MiSeq* and the *COVIDSeq Assay* (*96 Samples*) library preparation kit plus the *MiSeq Reagent Kit v2* (*300 cycles*). Bioinformatic analyses were costed using in-house computing for high throughput situations. Optimization Scenario: Substituted the *Illumina MiniSeq* instrument and the *Illumina COVIDSeq Test* (up to 3,072 samples) for library preparation with the *MiniSeq High Output Reagent Kit* (*300 cycles*) for sequencing. All other inputs remained identical to scenario A. Scale-up scenario: Using the same instrument as in the Validation Scenario but with a higher sample throughput of 5,000 samples/year and 2 runs/week. The library preparation was performed using the higher throughput *Illumina COVIDSeq Test* accompanied by the *MiSeq® Reagent Kit v2* (*300 cycle*). All scenarios a sequencing success rate of 85% was assumed^a^. For the ONT instrumentation for Laboratory 3: the Validation and Scale-up Scenarios used ONT MinION, and the Optimization Scenario used the ONT GridION. All 3 scenarios were costed using the ONT bundled sequencing reagents with the COVID Mini library preparation kit. The assumed sequencing success rate was 85% for all ONT scenarios^a^.

a Sequencing success rate must be estimated by the user reflecting level of expertise and previous experience.

b All costs are shown in United States dollars (USD).

### 2.5 Ethical approval

Consent to use the data from the pilot exercises was obtained from the participating laboratories. Additionally, a waiver was granted by the WHO Ethics Review Committee (Project ID - ERC.0003954) to publish the findings from this activity.

## 3 Results

During the GCT pilot exercises, all three laboratories provided the necessary information to calculate their current genomic sequencing costs (Validation Scenario) and to analyze two additional scenarios: a change in sequencing platform (Optimization Scenario) and an increase in annual sample throughput (Scale-up Scenario). Laboratories 1 and 2 use Illumina and Oxford Nanopore Technologies (ONT) platforms, respectively, and Laboratory 3 operates both platforms in parallel.

As expected, the total establishment costs,^1^ annual operational costs, and reagent and consumable costs increase as the annual throughput increases from 600 to 5,000 samples using the same equipment across all three laboratories (Tables 2, 3). The cost of the sequencing instrument alone, including 1 year of equipment maintenance, remains constant across all laboratories regardless of throughput.

In the Optimization Scenario, when analyzing cost per sample, Laboratory 1, using Illumina MiSeq, saw the average cost per sample decrease from USD 377 for 600 samples/year to USD 128 for 5,000 samples/year, a reduction of 66% (Table 2). Similarly, Laboratory 2, using ONT MinION, experienced a decrease from USD 456 to USD 101 per sample, a 78% reduction, (Table 2). Laboratory 3 showed an almost 80% reduction in cost per sample for Illumina MiSeq (from USD 1,162 to USD 234) and an 82% reduction for ONT MinION (from USD 963 to USD 173) when increasing throughput (Tables 2, 3).

**Figure 1:**
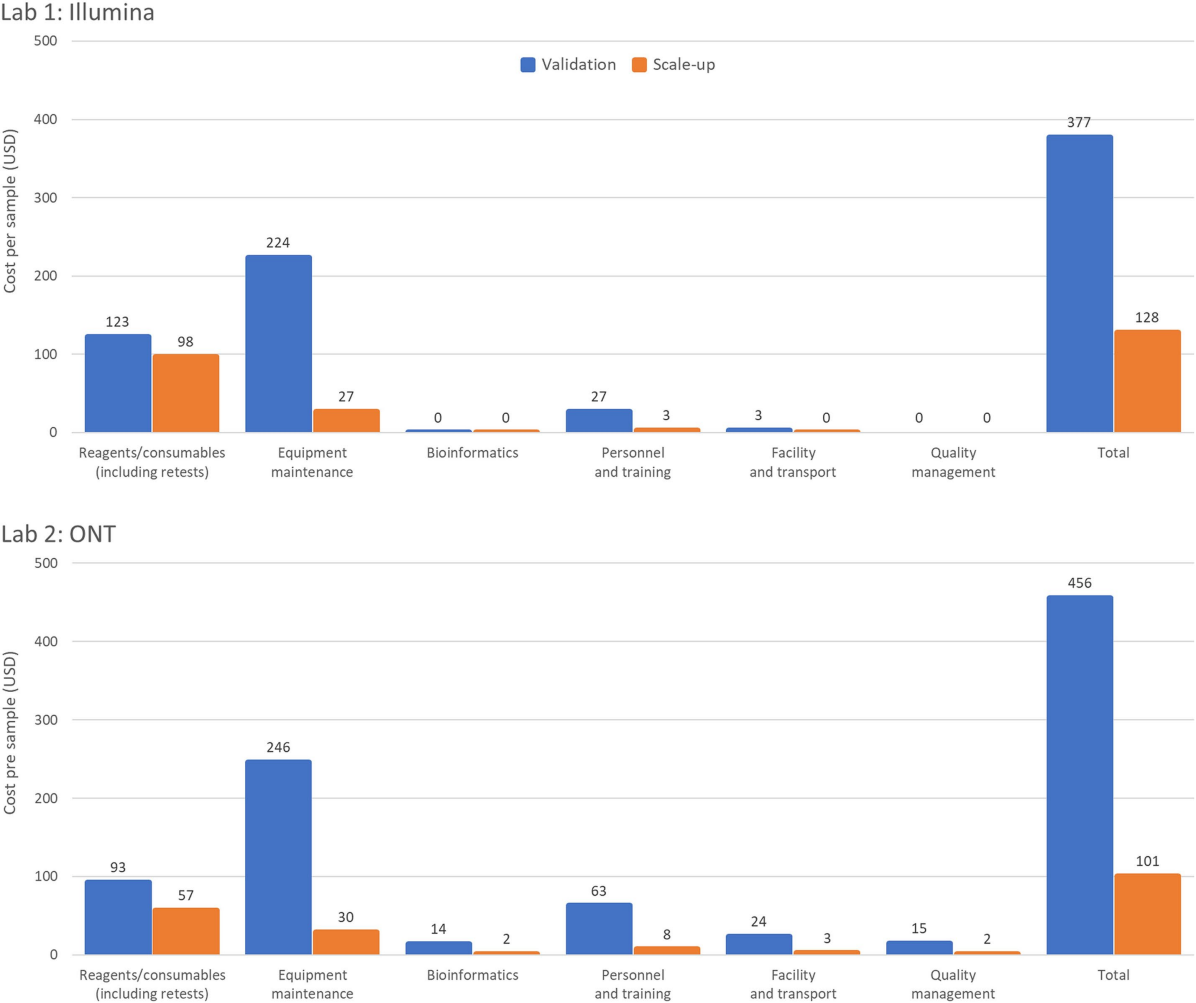
Summary of per sample costs with low- and high-throughput setups. The Validation Scenario calculated per sample costs based on an annual throughput of 600 samples with a single run performed each week over 36 weeks. Laboratory 1 (a lower-middle income country (LMIC) in the WHO European region) used an *Illumina MiSeq* and *EasySeq SARS-CoV-2 whole genome sequencing* (*WGS*) *Library Preparation Kit* (*96 samples*) plus the *MiSeq Reagent Kit v2* (*300 cycles*). Laboratory 2 (LMIC, WHO Africa region) calculations based on an *ONT MinION* instrument for using the *ONT bundled sequencing reagents* with the *COVID Mini* library preparation kit. The Scale-up Scenario used the same instrument as the Validation Scenario but with a sample throughput of 5,000 samples/year and 2 runs/week. Both laboratories’ bioinformatic analyses were costed using in-house computing for low throughput situations.

Workflow optimization could also involve a change in sequencing platforms, particularly when there is a significant shift in sample throughput or in the case of the availability of a more cost-efficient or fit-for-purpose platform or reagents. Laboratory 1 results (Table 2) showed that using an Illumina MiniSeq (a lower throughput instrument), instead of an Illumina MiSeq for an annual throughput of 600 samples, reduces the cost per sample by 3.3% (from USD 377 to USD 365). Similarly, in Laboratory 2, using a lower throughput ONT MinION instead of the ONT GridION reduces the cost per sample by 9% (from USD 497 to USD 456). Laboratory 3 (Table 3) also showed slight cost reductions when using lower throughput platforms for low annual throughput: Illumina from USD 1,162 to USD 1,143 (a 2% reduction) and ONT from USD 1,072 to USD 963 (an 11% reduction). The Scale-up Scenario demonstrated that an increase in annual sample numbers from 600 to 5,000 for these laboratories, led to an increase in the percentage of sequencer capacity used, causing a significant decrease in the reagent and consumable costs per sample. Laboratory 1 saw a 25.5% reduction (USD 123 to USD 98) Laboratory 2 experienced a 63.2% decrease (USD 93 to USD 57) and Laboratory 3 observed reductions of 49% with Illumina MiSeq (USD 258 to USD 126) and 53% with ONT MinION (USD 167 to USD 78). The differences in per-sample costs between low- and high-throughput setups, as derived from the Validation and Scale-up Scenarios, are visualized in Figure 1.

Equipment maintenance and calibration costs per sample decreased 88% across all laboratories and platforms as sample numbers increased, with costs dropping from USD 224 to USD 27 for Laboratory 1, and from USD 246 to USD 30 for Laboratory 2, and from USD 186 to USD 22 (Illumina MiSeq) or USD 79 to USD 10 (ONT MinION) for Laboratory 3 (Tables 2, 3). Maintenance costs can account for up to 59% of sequencing costs per sample across laboratories (Table 2).

## 4 Discussion

The GCT allows visualization of expected costs for sequencing activities, including equipment investment, maintenance, human resources, and quality management, which are not easily accessible elsewhere. This tool offers a transparent, reliable, and comprehensive assessment, enabling laboratories to formalize budget needs across various categories and compare sequencing costs. The GCT stands out from other costing tools due to its comprehensive scope (9). This flexibility ensures that the GCT supports objective cost analysis across diverse laboratory settings, without emphasizing comparisons between specific platforms. By focusing on a laboratory’s unique operational needs, the tool promotes equitable and context-specific decision-making. Pre-existing tools are limited to specific pathogens or single-cost categories, often lacking flexibility for scenario testing such as varied supplier and package sizes for sequencing items. The GCT covers establishment and operational costs, including equipment amortization, workforce expenses, and quality management. It is customizable to laboratory-specific parameters, allowing for scenario-based costing and optimization.

The pilot exercises highlighted the tool’s versatility, revealing significant variations in pricing of sequencing components based on geographical location. Notably, the total establishment costs for the Eastern Mediterranean region were nearly double those of the European and African regions. While the impact on initial investment on overall costs is generally minimal for low-throughput settings, it can be significant in high-throughput settings that require the purchase of additional ancillary equipment and automation to sustain the higher sequencing volumes. However, in these smaller throughput laboratories, not being able to efficiently fill sequencing run capacity makes sequencing costs even higher and can result in reagent waste. In addition, this context can also cause delays in the sharing of genetic sequence data, as laboratories often batch specimens to optimize resources. As the number of samples sequenced increases, the overall cost per sample, including equipment maintenance and consumables, decreases, demonstrating the cost-effectiveness of right-fit sequencing instrumentation, and the potential value of high-throughput platforms. This shows the inverse relationship between annual throughput and cost per sample, providing valuable insights for laboratories and policymakers.

The number of samples that can be added to a single run is determined by the loading capacity of the sequencing cartridge or flow cell and the output of the sequencing platform. Major cost efficiencies can be achieved by utilizing the maximum loading capacity of the instrument and sequencing kits or flow cells available in the laboratory. With a small number of samples sequenced per year the maximum loading capacity is not being utilized leading to less economic use of resources. Increasing the sample throughput from 600 to 5,000 samples per year resulted in an average fourfold increase in sequencer capacity across all laboratories, reaching a maximum of 99% loading capacity. This led to a significant reduction in cost per sample, although the magnitude of cost reduction differed slightly between Illumina and ONT platforms, depending on regional price differences for reagents ordered from certain manufacturers/suppliers. Figure 1 illustrates the profound impact of increasing sample throughput on reducing per-sample costs. This reinforces the value of optimizing laboratory workflows to maximize platform utilization, particularly in resource-constrained settings, where achieving cost-efficiency is critical for scaling genomic surveillance. Maximizing throughput to minimize costs per sample can be achieved by expanding genomic sequencing programs, establishing core sequencing facilities, broadening the range of pathogens studied, and enabling mixed sequencing runs.

There were notable disparities in price reduction due to increased throughput between Laboratories 1 and 3 on the Illumina MiSeq platform. This variation is attributed to platform-specific costs, such as bulk packaging for sequencing reagents, which can impact reagent waste and overall cost efficiency. As for Laboratory 3 a driver for higher costs were also procurement regulations which require the use of regional suppliers as an inevitable factor.

**Figure 2:**
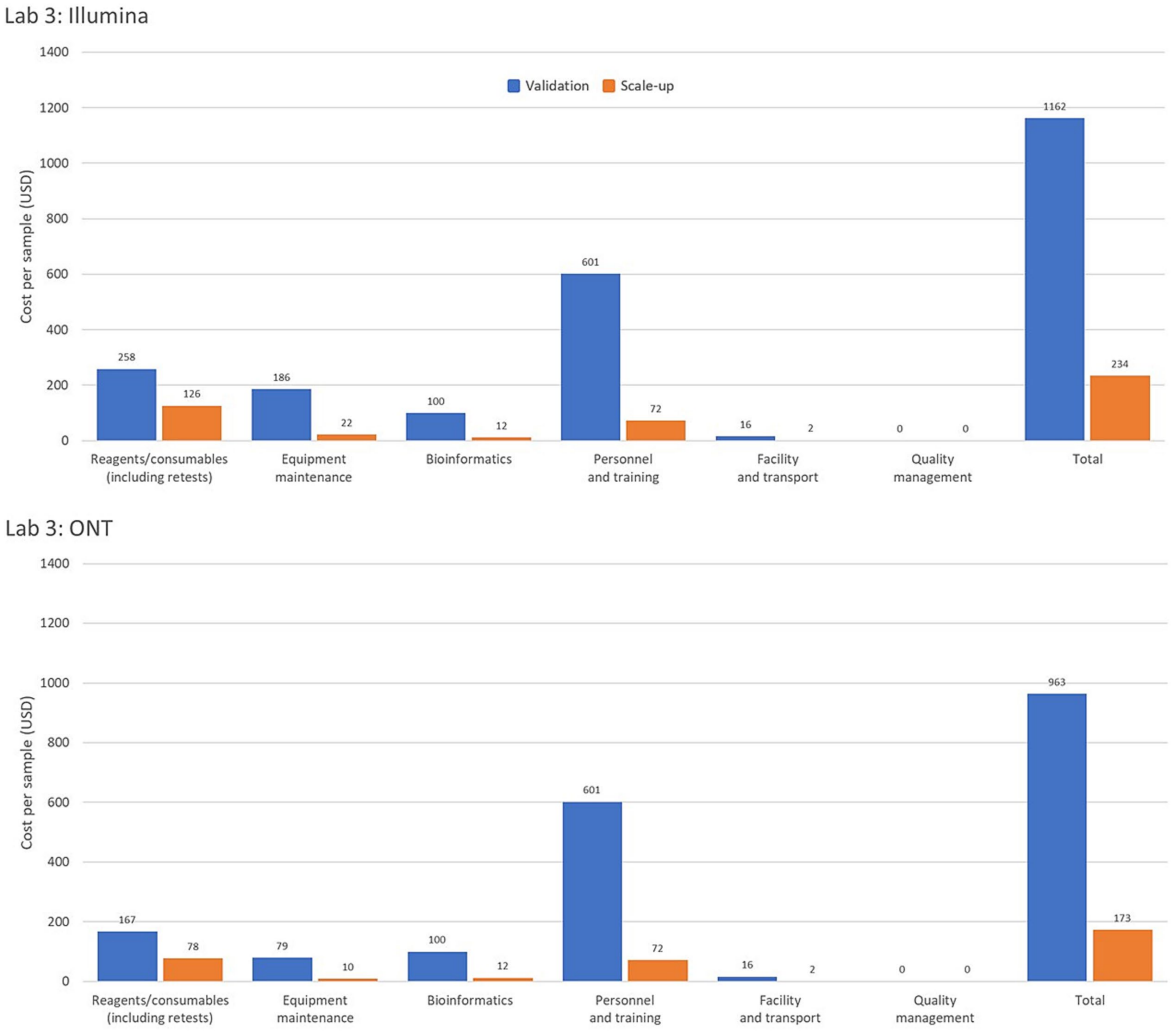
Comparison of genomic costing tool output data from a high-income country laboratory as per sample costing by operational category for the Illumina and Oxford Nanopore Technologies (ONT) platforms assessing low and high-throughput sample costing. The Validation Scenario calculated per sample costs based on an annual throughput of 600 samples with a single run performed each week over 36 weeks. Laboratory 3, from a high-income country in the WHO Mediterranean region, based calculations on an *Illumina MiSeq* and *EasySeq SARS-CoV-2 whole genome sequencing* (*WGS*) *Library Preparation Kit* (*96 samples*) plus the *MiSeq Reagent Kit v2* (*300 cycles*). The ONT platform calculations were for the *ONT MinION* instrument using the *ONT bundled sequencing reagents* with the *COVID Mini* library preparation kit. The Scale-up Scenario used the same instrument as the Validation Scenario with a sample throughput of 5,000 samples/year and 2 runs/week. Bioinformatic analyses were costed using in-house computing for low throughput situations.

Interestingly, one of the pilot laboratories uses two automated extraction instruments for 600 samples. If the laboratory were to use manual extraction instead, the equipment maintenance and calibration cost per sample would decrease by 23.9% (from USD 246 to 200) and the overall cost per sample would decrease by 11.2% (from USD 456 to 410). This underscores the importance of evaluating the feasibility and cost implications of acquiring or accepting donations of expensive equipment, particularly those requiring substantial resources for operation and maintenance. Figure 2 provides a comparison of per-sample costs by operational category for Illumina and ONT platforms in Laboratory 3, highlighting cost variations under low- and high-throughput conditions.

**Figure 3:**
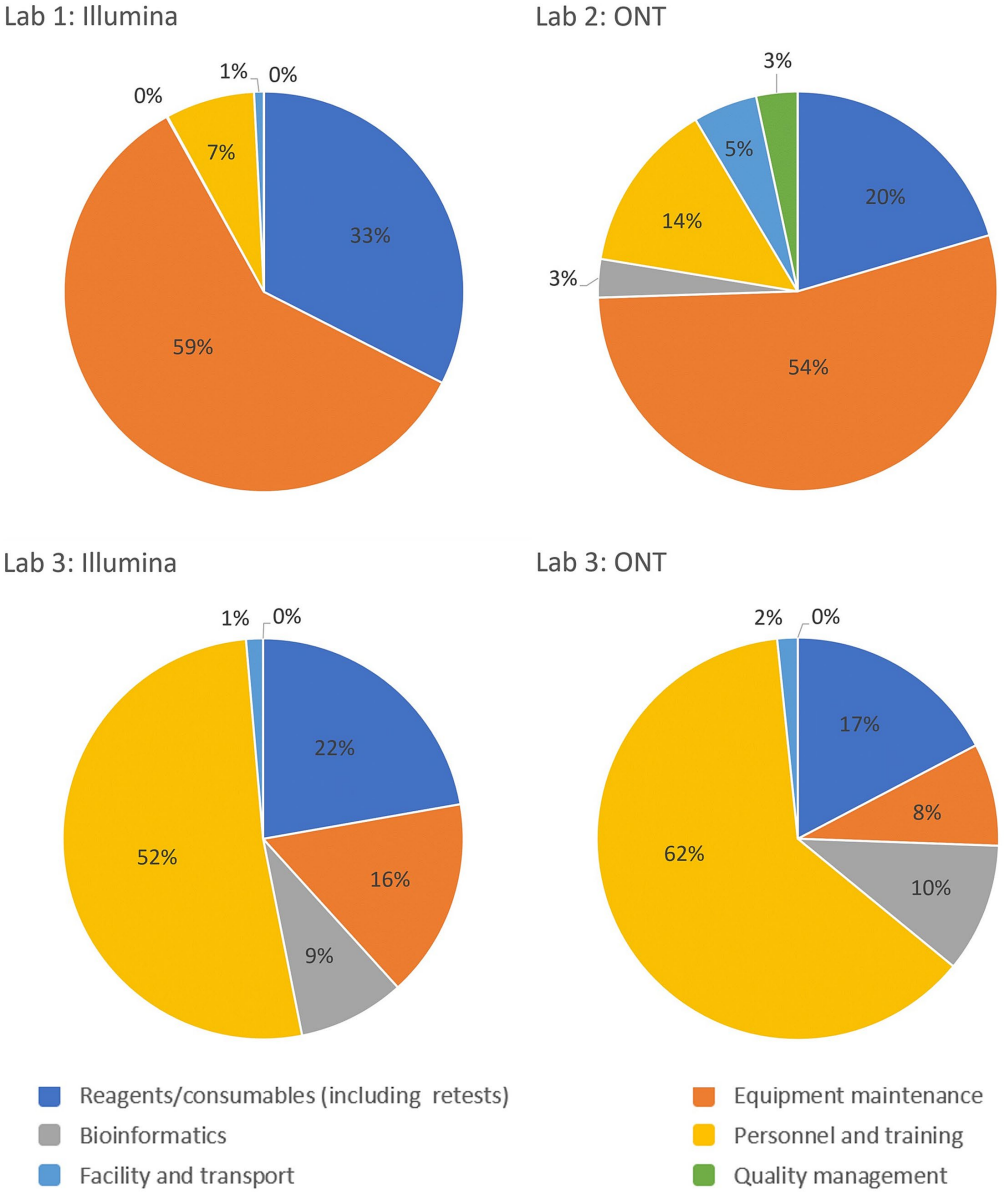
Breakdown of the cost drivers by platform and operational category as a percentage of costs per sample for the Validation Scenario in each laboratory. Laboratory 1 and 2 are lower-middle income countries in the European and African WHO regions respectively and Laboratory 3 represents a high-income country from the Eastern Mediterranean WHO region.

Reagents and consumables are often perceived as major drivers of sequencing costs, but the GCT revealed that they account for only 16–33% of the costs per sample. This highlights the need for comprehensive costing that includes equipment, personnel and training, facilities, transport, bioinformatics, and quality management systems. A breakdown of cost drivers as a percentage of per-sample costs for each laboratory is illustrated in Figure 3, highlighting variations across the three pilot laboratories and sequencing platforms. This underscores the importance of analyzing key cost components such as equipment maintenance, calibration, and reagents, which can vary significantly based on regional and platform-specific factors. The charts highlight the need for tailored cost-optimization strategies that address these differences to enhance the sustainability of sequencing programs across diverse settings. The GCT addresses these costs in detail. Given the specific training required for sequencing, countries, and programs must consider initial and ongoing training costs and budget for competitive salaries to retain trained staff, especially in low- and middle-income countries, where staff turnover is high due to low salaries. Offering competitive salaries and implementing contractual regulations are essential for sustaining investments in human resources.

When evaluating the investment costs, GCT allows for projecting costs according to different sequencing platforms and equipment. Costs will vary based on equipment capacity and the actual number of samples tested. Therefore, a realistic assessment of sample volumes is needed to select the appropriate platform. Low sample throughput leads to uneconomic costs per sample if constant or real-time sequencing output is required such as often the case in public health laboratories. Gradual capacity building with lower throughput equipment before investing in higher throughput platforms, or centralizing sequencing capacities to maximize throughput and cost efficiency, are strategies for optimizing costs. Maintenance costs are a significant component of running costs and must be factored into laboratory budgets to ensure sustainable services. As shown in Figure 2, operational costs for Illumina and ONT platforms in a high-income country laboratory reveal significant variations in cost-efficiency between platforms and throughput levels. These insights provide valuable guidance for laboratories and policymakers in selecting platforms and structuring operations to achieve optimal cost-effectiveness.

It is in a country’s interest to integrate sequencing capacity for other high-priority pathogens to reduce investment and running costs, making more efficient use of existing capacities. The GCT provides insights into sustainability, stability, and better utilization of the funding, offering guidance to donors when procuring sequencing equipment based on laboratory capacity and projections.

Integrating the GCT into ongoing genomic initiatives requires strategic alignment with operational priorities, funding frameworks, and partnerships. Institutionalizing the tool through integration into national genomic strategies can inform annual procurement plans for reagents, equipment, and staffing. The GCT allows cross-sector applications as it can be employed in agriculture, veterinary sciences, and environmental monitoring to support a broader One Health approach and be an important component of pandemic preparedness plans, the GCT can estimate costs for rapidly scaling genomic surveillance during outbreaks, enhancing responsiveness. The tool provides the footing for evidence-based advocacy for sustainable funding. Using GCT data, initiatives can be embedded into national health budgets as part of broader disease control and preparedness programs. Ministries of Health can leverage cost analyses to advocate for domestic funding. Data from the GCT can also help justify resource allocation by demonstrating the cost-effectiveness and impact of genomic surveillance. For instance, detailed cost breakdowns can support funding proposals to governments, international organizations, or donor organizations. The GCT can help design innovative financing models, combining public, private, and philanthropic funds. For instance, private entities might co-invest in genomics initiatives if the tool demonstrates shared benefits (e.g., for pharmaceutical research and development or supply chain optimization). Partnerships with global health philanthropic organizations could help address capacity gaps identified by the GCT, like training bioinformaticians or equipping labs. The GCT could be used to encourage collaborations between governments, biotech companies, and academic institutions by providing clarity on financial contributions and cost-sharing models. By driving evidence-based resource allocation, fostering partnerships, and enabling cost-efficient scaling, the GCT could be a cornerstone of sustainable genomic surveillance initiatives.

During the pilot exercises, most laboratories were sequencing pathogens beyond SARS-CoV-2, including influenza viruses, drug-resistant *Mycobacterium tuberculosis*, and *Escherichia coli*. This is particularly relevant for laboratories initially established to sequence SARS-CoV-2 during the pandemic and have since transitioned to include other pathogens in their sequencing workflows. In addition to expanding the range of pathogens, accommodating diverse sample types and adopting broader genomic approaches, such as sequencing panels, is increasingly important. Efforts are underway to integrate sequencing programs across multiple pathogens to sustain advancements in genomic sequencing, with plans to expand the use of the GCT to other pathogens and sequencing platforms in 2025.

Economic efficiency and fair access to genomic resources must be balanced to address the ethical aspects of prioritizing pathogens or geographical areas according to cost-effectiveness as these decisions have broad policy implications that can impact equity, resource allocation, and public trust. Cost-effectiveness metrics might favor regions or pathogens with existing infrastructure or higher throughput capabilities, potentially marginalizing under-resourced areas or neglected diseases. Decisions should consider the broader health and equity impacts, such as prioritizing regions with higher disease burdens or populations with limited healthcare access. Policymakers should incorporate health impact assessments into decision-making to ensure comprehensive surveillance beyond direct cost savings. Costs along with equity, public health urgency, and regional needs need to be integrated into policy frameworks and funding strategies. The GCT can be a tool that not only optimizes costs but also promotes fairness and inclusivity in global genomic surveillance efforts.

## 5 Conclusion

The GCT offers essential cost information for establishing and maintaining sequencing laboratories, supporting critical budget planning and optimization of sequencing workflows as well as improving timelines for sharing genomic sequence data. By highlighting key cost drivers in sequencing workflows, it helps guide the procurement and placement of sequencing platforms and resources based on annual throughput and associated costs. The inclusion of proportional investment costs allows users to evaluate cost efficiency across scenarios, ensuring relevance for laboratories in varying economic and operational conditions. This supports the GCT’s role in guiding evidence-based budgeting and sustainability planning. When a laboratory needs to scale up its sequencing program, the tool can estimate costs for various scale-up scenarios, considering projected annual sample throughput, facility, and workforce expenses. This forecasting approach is valuable for developing national genomic strategies, with the GCT providing crucial insights.

## Group members of GCT pilot working group

Anita Suresh, Genomics and Sequencing, FIND, Switzerland; Beatrix Kele, Virus Reference Department, United Kingdom Health Security Agency, London, United Kingdom; Bright Adu, Department of Immunology, Noguchi Memorial Institute for Medical Research, University of Ghana, Ghana, Christopher Sunkwa Tamal, Emergency Preparedness and Response, World Health Organization, Ghana; Gifty Boateng, National Public Health and Reference Laboratory, Ghana Health Service, Ghana; Gulmira Kalmambetova, National Tuberculosis Center, National TB Reference Laboratory, Bishkek, Kyrgyzstan; Hanan Al Kindi, Central Public Health Laboratories, Centre for Disease Control and Prevention, Ministry of Health, Muscat, Oman; Kwame Asante, Association of Public Laboratories, Accra, Ghana; Leena Inamdar, New Variant Assessment Platform, UK Health Security Agency, London, United Kingdom; Luke Meredith, Infectious Hazard Prevention and Preparedness, World Health Organization Regional Office for the Eastern Mediterranean, Cairo, Egypt; Nicksy Gumede-Moeletsi, Emergency Preparedness and Response, World Health Organization Regional Office for Africa, Brazzaville, Congo; Rajesh Kumar, Association of Public Health Laboratories, Muscat, Oman; Swapna Uplekar, Genomics and Sequencing, FIND, Switzerland; Yathrib Mohamed Al Zakwani, Central Public Health Laboratories, Centre for Disease Control and Prevention, Ministry of Health, Muscat, Oman.

## Data Availability

The original contributions presented in the study are included in the article/Supplementary material, further inquiries can be directed to the corresponding author.

## Footnotes

^1^Total establishment costs: The total cost to establish and run a sequencing laboratory, including purchasing of new equipment as well as reagents and consumables for the first year.
