# Supplementary material for: Genomics costing tool: considerations for improving cost-efficiencies through cross scenario comparison
Source: Front Public Health. 2025 Jan 15;12:1498094. doi: 10.3389/fpubh.2024.1498094 (PMC11775897; doi:10.3389/fpubh.2024.1498094)
Supplement: Supplementary file 1 [file Data_Sheet_1.pdf]

NGS Costing Tool: Pre-Costing Site Readiness Questionnaire

User Profiles of NGS Costing Tool

#1: Laboratories sequencing samples collected through national surveillance initiatives;

#2: Laboratories wishing to expand, optimise, or cost their NGS services;

#3: Health policy stakeholders wishing to evidence timelines to build/adapt new NGS services in a country or region.

Objectives of Pre-Costing Questionnaire

#1: Determine laboratory's costing needs can be met through use of NGS Costing Tool, by gaging whether laboratory requesting to use the tool:

- has ad-hoc NGS projects they wish to fund,
- is trying to cost routine NGS activities/projects, or is trying to validate already costed NGS activities/projects,
- wishes to obtain baseline of current capacity and services that can be compared after use of tool to implement change to NGS activities,
- aims to streamline the current purchasing in place by more clearly showcasing all available supplies across different sequencing platforms/workflows.

#2: Gather the minimum information required by the trainer of NGS costing tool to allow efficient implementation of the costing tool

Description of NGS Costing Tool Outputs

The NGS Costing tool can offer detailed breakdown of costs for molecular labs wishing to start or expand sequencing activities for SARS-CoV-2, as per cost categories detailed below:

SETUP & RUNNING EXPENSES FOR GENERAL MOLECULAR LAB

- Lab building infrastructure (ex. CL2, CL3, etc), needed equipment & supplies, and facility running costs (maintenance cost, routine supply ordering)
- Sample transport, receipt, inactivation, and processing
- Sample extraction and PCR

SETUP & RUNNING EXPENSES FOR SEQUENCING LAB (for low vs high weekly throughputs)

- Library prep/pooling
- Sequencing
- Bioinformatics/Data analysis (ex. pipeline costs) and IT infrastructure (ex. data storage costs)
- Molecular PCR and Sequencing EQA

PERSONNEL COSTS

- Personnel cost based on time spent on NGS activities (for sample sequencing, for bioinformatics analysis, and for routine reporting) & training needs

Site Readiness Questionnaire:

PART 1: BASELINE INFORMATION ABOUT LABORATORY

1) INSTITUTION PROFILE: Please answer the two questions below regarding the general profile of your laboratory

A. Please specify the type of NGS laboratory

☐ International reference lab (i.e. receiving samples from other countries)
☐ National reference lab (i.e. receiving all surveillance samples in the country)
☐ Sub-National reference lab (i.e. receiving samples from a region in the country)
☐ Other, please specify: _______________

B. Please select all affiliations that apply:

☐ Public health lab
☐ Hospital
☐ University lab
☐ Other, please specify: _______________

2) PATHOGENS AT YOUR LAB: Please list all priority pathogens that are tested for at your lab as per categories below:

Pathogens under PCR testing only (i.e. those tested under either routine or targeted surveillance activities)	Pathogens under NGS detection (i.e. those sequenced under either routine or targeted surveillance activities)

(optional) Does your lab also conduct metagenomics when needed? ☐ yes ☐ no

3) NGS CAPACITY: Please answer the three questions below regarding the sequencing capacity of your laboratory

A. What was your lab's sequencing capacity (volume sequencing per month) of SARS-CoV-2 samples at peak of pandemic?
(Please enter number below)
______Desired number sequenced genomes per month
______Actual Number of sequenced genomes per month

B. Please specify below the NGS sequencing instrument(s) used in your lab – and which pathogens are sequenced using the instrument?

Name of Sequencing Instrument	Name of Pathogen being sequenced

C. Please specify current sequencing workflow from options below:

☐ Workflow 1 (automated lib prep &automated extraction- high throughput)

☐ Workflow 1 (automated lib prep only - high throughput)

☐ Workflow 2 (manual - high throughput)

☐ Workflow 3 (manual – low throughput)

4) NGS WORKFORCE: Post pandemic, what is the size your current NGS workforce?
(Please specify number for each category below):

__________ (# of lab scientists doing NGS)

___________(# of bioinformaticians)

5) ORDER PLACEMENT: Please answer the three questions below around lab supplies order placement at your institution:

A. Are routine lab supplies ordered through:

☐ external department (e.g. Ministry) ☐ internally by NGS lab staff

☐ donor or implementing partner ☐ other: ____________

B. Does your institution currently have challenges accessing the needed reagents and NGS supplies for existing platforms/workflows – due to challenges such as national procurement restrictions in supplier selection, access to needed packaging size, etc

☐ yes ☐ no

If yes, please specify the challenges: _________________

C. Experience level rating: For the person currently in charge of placing order of sequencing supplies for laboratory, please rate their current level of experience in placing needed orders for routine and new needed supplies – with 1 being little experience

☐ 1 - Little experience ☐ 2- some experience ☐ 3- very experienced

6) TRAINING NEEDS: Do you need additional trained personal to carry out sequencing activities?

☐ Not needed
☐ Epidemiologists
☐ Bioinformaticians
☐ Laboratory Scientists
☐ Other, please specify

7) **FINANCIAL FLOWS - How is your genomic surveillance programme currently funded – please answer below:**

A. Funding source (*e.g. WHO Emergency funding, Government funding*) :_______________

B. Has future NGS funding envelope for next 1-2 years been secured?

☐ yes ☐ no ☐ I'm not sure

C. Is your lab project expenditure timeline tracked

☐ quarterly ☐ annually

☐ bi-annually ☐ not regularly tracking

☐ other (please specify):_____

D. Please detail the cost categories for expenses routinely tracked at your laboratory

☐ Laboratory running expenses
☐ QA & accreditation
☐ Computing infrastructure,
☐ logistics and storage,
☐ Personnel and training needs
☐ LIMS licensing & upkeep
☒ Other: please specify: _______________

☐ N/A

PART 2: COSTING TOOL PRE ASSESSMENT

8) **COSTING SCENARIOS – Please specify from scenarios below what your lab wishes to cost regarding current or upcoming NGS activities**

☐ (Scale-up Scenario #1) I wish to Inform MoH policy makers that want to grow/expand genomic services (full cost of setting up and annual running cost of N-PHL to do NGS sequencing)

☐ (Scale-up Scenario #2) I wish to determine the overhead costs to scale up my lab from lower throughput to higher throughput – and the size/time allocation of workforce and automation required at the higher throughput where applicable

☐ (Optimising Scenario) I wish to make informed decisions on how to get better value for money for sequencing activities by optimising sequencing workflows, to stretch my budget for NGS

☐ (Validation Scenario) I wish to validate a recent costing exercise conducted at my lab for NGS activities

☐ Other: _________ (please specify)

9) **EXISTING COSTING TOOLS – Please answer the two questions below regarding any costing tools currently in use at your institution:**

A. Please select which tool is used for completing costing of scenarios as per above:

☐ Excel spreadsheet
☐ Manual calculation *(ex. cost estimates explained in a document)*

☐ Lab-customised costing tool
☐ Others (please specify): ___________
☐ None

B. How challenging is it to currently to conduct a costing exercise, using your existing tools? (please select one below):

(1) Easy	(2) Moderate	(3) Challenging	(4) Very challenging
☐	☐	☐	☐

10) COSTING OF NATIONAL GENOMIC SURVEILLANCE INITIATIVES: Please answer the remaining questions below regarding your lab's inputs into national plans for genomic surveillance:

A. Is there a SARS-CoV-2 national surveillance strategy action plan (e.g. NAPHS) currently being implemented in your country:
☐ yes -with genomic surveillance included
☐ yes -without genomic surveillance included
☐ no
☐ unsure

B. If yes to question directly above – does the plan include a budget for genomic surveillance?
☐ yes ☐ no ☐ unsure

C. If yes to question directly above, which department in your national program is responsible for overall costing of genomic sequencing preparedness for newly emerging high-threat pathogens?
(please specify department name below):
____

D. Are costing tools from your institution provided as inputs to this process?

☐ yes, my institution provides inputs to this process

☐ no, my institution does not provide inputs

☐ N/A: no costing tool implemented

E. If yes to question directly above, do you account for changes in laboratory workforce/personnel in this process?

☐ yes ☐ no ☐ N/A

F. Do you require access to a more detailed laboratory costing tool to forecast enhancement of future services?
☐ yes ☐ no

Annex 1: List of required inputs to prepare ahead of NGS Costing Tool Completion

1) Number of SARS-CoV-2 samples sequenced last year
2) Platform used for sequencing of SARS-CoV-2
3) List of reagents and consumables used for sequencing of SARS-CoV-2 with prices (from sampling to analyzing data)
4) Estimated quantity of those reagents and consumables used in the last year
5) List of equipment and software used for bioinformatics for SARS-CoV-2 with prices
6) List of all maintenance contracts for sequencing equipment with prices
7) List of all the equipment used for sequencing of SARS-CoV-2 (from sampling to analyzing data) with prices
8) List of all personnel involved in sequencing and bioinformatics with salary and training costs in the last year
9) List of all facility-related costs with prices for the last year (electricity, internet, heating water, telephone waste management, etc.)
10) Costs for transportation related to sequencing last year
11) Costs for QMS (accreditation, EQAs, etc) for sequencing purposes last year
