# Supplementary material for: Genomics costing tool: considerations for improving cost-efficiencies through cross scenario comparison
Source: Front Public Health. 2025 Jan 15;12:1498094. doi: 10.3389/fpubh.2024.1498094 (PMC11775897; doi:10.3389/fpubh.2024.1498094)
Supplement: Supplementary file 2 [file Data_Sheet_2.pdf]

Post pilot survey tool

Thank you for participating in the pilot exercise for the genomics costing tool. We would like to get your feedback on the use of the tool. This survey will take less than 8 minutes of your time.

1. How easy was it for you to understand how the tool works?
   1- Very difficult
   2- Difficult
   3- Neutral
   4- Easy
   5- Very easy

Any comments:

2. How easy was the tool for you to use?
   1- Very difficult
   2- Difficult
   3- Neutral
   4- Easy
   5- Very easy

Any comments:

3. How valuable do you think the tool is to your lab?
   1- Not valuable at all
   2- Not very valuable
   3- Neutral
   4- Valuable
   5- Very valuable

Any comments:

4. How long did it take you to complete the tool for the costing scenario that validates cost in your lab?
   1- > 8 hours (more than one working day)
   2- 6 – 8 hours
   3- 3 – 5 hours
   4- 1 – 3 hours
   5- < 1 hour

Any comments:

5. What do you think about the completion time?
   1- Very long
   2- Long
   3- Neutral
   4- Short
   5- Very short

Any comments:

6. Would you rather have a web-based tool?
   1- Yes
   2- No
   3- I would like to have both options

Any comments:

7. How likely are you to use the tool for future projections?
   1- Never
   2- Unlikely
   3- Neutral
   4- Likely
   5- Very likely

Any comments:

8. How likely are you to recommend this tool to your colleagues?
   1- Never
   2- Unlikely
   3- Neutral
   4- Likely
   5- Very likely

Any comments:

9. Please rank the potential use of the tool in your lab from options below

   ___Grant applications

   ___Budgeting for routine sequencing and bioinformatics activities

   ___Budgeting for sequencing and bioinformatics activities as an international referral lab

   ___Cost optimization

   ___Retrospective costing

   ___Others: please specify

10. Was there anything missing in the tool that should be added or changed?
11. Other comments
